# A familial FTD associated with *C9orf72* repeat expansion and dysplastic gangliocytoma

**DOI:** 10.1186/1750-1326-8-S1-P60

**Published:** 2013-10-04

**Authors:** Mia Kero, Raffaele Ferrari, Kin Mok, Anders Paetau, Pentti J Tienari, Olli Tynninen, John Hardy, Parastoo Momeni, Auli Verkkoniemi-Ahola, Liisa Myllykangas

**Affiliations:** 1 Laboratory of Neurogenetics, Department of Internal Medicine, Texas Tech University Health Sciences Center, Lubbock, TX, USA; 2 Institute of Neurology, University College London, Queen Square, London, UK; 3 Departments of Pathology, Haartman Institute, University of Helsinki and HUSLAB,, Helsinki, Finland; 4 Research Program for Molecular Neuroscience, Biomedicum Helsinki, Helsinki, Finland; 5 Department of Clinical Neuroscience Helsinki University Central Hospital, Helsinki, Finland; 6 Folkhälsan Institute of Genetics, Biomedicum Helsinki, Helsinki, Finland

## Background

A hexanucleotide repeat expansion, in the chromosome 9 open reading frame 72 gene (*C9orf72*), has been identified the most common genetic cause of FTD/ALS. Here we describe the clinical, pathologic and genetic features of a Finnish *C9orf72* expansion carrier, who developed a dysplastic gangliocytoma, a rare hamartoma of cerebellar granule cells, associated with *PTEN* mutations. In addition to the dysplastic gangliocytoma, the patient showed TDP43-pathology in the cortex and in the substantia nigra, and p62-positive/TDP43-negative inclusions in the cerebellar granule cells. His sister carried the same gene defect and showed similar type of TDP43/p62-pathology in her brain. Our findings confirm the clinical and pathological picture of *C9orf72* mutation carriers is more heterogeneous than originally thought and warrant further studies on the possible involvement of PTEN pathway in the specific cerebellar granule cell pathology associated with *C9orf72* expansion.

## Materials and methods

The index case and his sister were diagnosed with FTD at the Helsinki Central University Hospital. Blood samples for DNA analysis were collected from the index patient and his 4 siblings, and 4 other family members. The neuropathological examination and sample collection of the index patient and his diseased sister was performed using standardised methods. In addition tohaematoxylin-eosin staining , IHC against TDP-43, p62, ubiquitin, and tau proteins was performed. The candidate genes (*MAPT*), *PGRN* and *TDP-43* were sequenced in 5 siblings. Presence of repeat expansion in *C9orf72* was investigated by RP-PCR in the 5 siblings. All reactions were run on an ABI3730 DNA Analyzer and the results were processed using Sequencher 4.9 and Gene Mapper software. The range of ≥40 repeats defined as the threshold to discriminate presence vs. absence of expansion in *C9orf72*. For the index patient, *PTEN* gene was sequenced and MPLA was performed, as this gene has been found mutated in the majority of patients with adult-onset dysplastic gangliocytomas.

## Results

In addition to the dysplastic gangliocytoma, the index patient, and her sister showed TDP43-pathology mainly in the cortex and in the substantia nigra, and p62-positive/TDP43-negative inclusions in the cerebellar granule cells. Sequencing of the candidate genes *MAPT*, *PGRN* and *TDP-43* did not reveal coding variants in any of the subjects. Sequencing and MLPA analysis of the *PTEN* gene did not show any mutations in the coding sequence. In the *C9orf72* rp-pcr analysis, the two diseased family members and one sibling did show a number of repeats (>40 repeats) above the presumed pathogenic range.

## Conclusions

Our findings confirm the clinical and pathological picture of *C9orf72* mutation carriers is more heterogeneous than originally thought. One might speculate that the presence of the *C9orf72* mutation might have induced the PTEN depletion in the cerebellar granule cells, which had resulted in the development of dysplastic gangliocytoma in this individual.

